# Genetic analysis of hair samples attributed to yeti, bigfoot and other anomalous primates

**DOI:** 10.1098/rspb.2014.0161

**Published:** 2014-08-22

**Authors:** Bryan C. Sykes, Rhettman A. Mullis, Christophe Hagenmuller, Terry W. Melton, Michel Sartori

**Affiliations:** 1 Institute of Human Genetics, Wolfson College, University of Oxford, Oxford OX2 6UD, UK; 2 PO Box 40143, Bellevue, WA 98005, USA; 3 NaturAlpes, Annecy-Le-Vieux 74940, France; 4 Mitotyping Technologies, 2565 Park Center Boulevard, State College, PA 16801, USA; 5 Museum of Zoology, Palais de Rumine, Lausanne 1014, Switzerland; 6 Museum of Zoology and Grindel Biocentre, Hamburg 20146, Germany

**Keywords:** yeti, almasty, bigfoot, sasquatch, mitochondrial DNA

## Abstract

In the first ever systematic genetic survey, we have used rigorous decontamination followed by mitochondrial 12S RNA sequencing to identify the species origin of 30 hair samples attributed to anomalous primates. Two Himalayan samples, one from Ladakh, India, the other from Bhutan, had their closest genetic affinity with a Palaeolithic polar bear, *Ursus maritimus*. Otherwise the hairs were from a range of known extant mammals.

## 1. Introduction

Despite several decades of research, mystery still surrounds the species identity of so-called anomalous primates such as the yeti in the Himalaya, almasty in central Asia and sasquatch/bigfoot in North America. On the one hand, numerous reports including eye-witness and footprint evidence, point to the existence of large unidentified primates in many regions of the world. On the other hand, no bodies or recent fossils of such creatures have ever been authenticated. There is no shortage of theories about what these animals may be, ranging from surviving populations of collateral hominids such as *Homo neanderthalensis*, *Homo floresiensis* [1] or Denisovans [2], extinct apes such as *Gigantopithecus* [3] or even unlikely hybrids between *Homo sapiens* and other mammals [4]. Modern science has largely avoided this field and advocates frequently complain that they have been ‘rejected by science’ [5]. This conflicts with the basic tenet that science neither rejects nor accepts anything without examining the evidence. To apply this philosophy to the study of anomalous primates and to introduce some clarity into this often murky field, we have carried out a systematic genetic survey of hair samples attributed to these creatures. Only two ‘tongue-in-cheek’ scientific publications report DNA sequence data from anomalous primates. Milinkovitch *et al*. [6], after analysis of a Nepalese sample, confirmed Captain Haddock's suspicions that the yeti was an ungulate [7]. The same conclusion was reached by Coltman *et al*. [8] after analysis of sasquatch hair from Alaska.

## 2. Material and methods

Hair samples submissions were solicited from museum and individual collections in a joint press release issued on 14 May 2012 by the Museum of Zoology, Lausanne and the University of Oxford. A total of 57 samples were received and subjected to macroscopic, microscopic and infrared fluorescence examination to eliminate obvious non-hairs. This excluded one sample of plant material and one of glass fibre. Of the screened samples, 37 were selected for genetic analysis based on their provenance or historic interest. Lengths (2–4 cm) of individual hair shaft were thoroughly cleaned to remove surface contamination, ground into a buffer solution in a glass homogenizer then incubated for 2 h at 56°C in a solution containing proteinase K before extraction with phenol/chloroform/isoamyl alcohol. PCR amplification of the ribosomal mitochondrial DNA 12S fragment corresponding to bps 1093–1196 of the human mitochondrial genome was carried out [9,10]. Recovered sequences were compared to GenBank accessions for species identification.

## 3. Results and discussion

The table 1 shows the GenBank species identification of sequences matching the 30 samples from which DNA was recovered. Seven samples failed to yield any DNA sequences despite multiple attempts. As the sequence of mitochondrial 12S RNA segment is identical in *H. sapiens* and *H. neanderthalensis*, amplification and sequencing of mitochondrial DNA hypervariable region 1 (bps 16 000–16 400) of no. 25072 was carried out and identified the source as being identical to the revised Cambridge Reference Sequence [11] and thus *H. sapiens* of likely European matrilineal descent. Other submitted samples were of known mammals that in most cases were living within their normal geographical range, the exceptions being sample nos. 25025 and 25191 (*Ursus maritimus*, polar bear) from the Himalayas, no. 25074 (*Ursus americanus*, American black bear) and no. 25075 (*Procyon lotor*, raccoon) that were submitted from Russia even though they are native to North America.

**Table 1.** Origin and GenBank sequence matches of hair samples attributed to anomalous primates. (All sequence matches were 100%.)

ref. no.	location	attribution	GenBank sequence match	common name
25025	Ladakh, India	*yeti*	*U. maritimus*	polar bear
25191	Bhutan	*yeti/migyhur*	*U. maritimus*	polar bear
25092	Nepal	*yeti*	*Capricornis sumatraensis*	serow
25027	Russia	*almasty*	*U. arctos*	brown bear
25039	Russia	*almasty*	*Equus caballus*	horse
25040	Russia	*almasty*	*Bos taurus*	cow
25041	Russia	*almasty*	*Equus caballus*	horse
25073	Russia	*almasty*	*Equus caballus*	horse
25074	Russia	*almasty*	*U. americanus*	American black bear
25075	Russia	*almasty*	*P. lotor*	raccoon
25194	Russia	*almasty*	*U. arctos*	brown bear
25044	Sumatra	*orang pendek*	*Tapirus indicus*	Malaysian tapir
25035	AZ, USA	*bigfoot*	*P. lotor*	raccoon
25167	AZ, USA	*bigfoot*	*Ovis aries*	sheep
25104	CA, USA	*bigfoot*	*U. americanus*	American black bear
25106	CA, USA	*bigfoot*	*U. americanus*	American black bear
25081	MN, USA	*bigfoot*	*Erethizon dorsatum*	N. American porcupine
25082	MN, USA	*bigfoot*	*U. americanus*	American black bear
25202	OR, USA	*bigfoot*	*U. americanus*	American black bear
25212	OR, USA	*bigfoot*	*C. lupus/latrans/domesticus*	wolf/coyote/dog
25023	TX, USA	*bigfoot*	*Equus caballus*	horse
25072	TX, USA	*bigfoot*	*Homo sapiens*	human
25028	WA, USA	*bigfoot*	*U. americanus*	American black bear
25029	WA, USA	*bigfoot*	*C. lupus/latrans/domesticus*	wolf/coyote/dog
25030	WA, USA	*bigfoot*	*Bos taurus*	cow
25069	WA, USA	*bigfoot*	*Odocoileus virginianus/hemionus*	white-tailed/mule deer
25086	WA, USA	*bigfoot*	*Bos taurus*	cow
25093	WA, USA	*bigfoot*	*C. lupus/latrans/domesticus*	wolf/coyote/dog
25112	WA, USA	*bigfoot*	*Bos taurus*	cow
25113	WA, USA	*bigfoot*	*C. lupus/latrans/domesticus*	wolf/coyote/dog

Despite the wide range of age and condition of the submitted hair shafts, which ranged from fresh to museum specimens more than 50 years old, the majority yielded mitochondrial 12S RNA sequences which allowed species identification with 100% sequence identity. Of the recovered sequences, only one (no. 25072) yielded a human sequence, indicating that the rigorous cleaning and extraction protocol had been effective in eliminating extraneous human contamination which often confounds the analysis of old material and may lead to misinterpretation of a sample as human or even as an unlikely and unknown human x mammalian hybrid [4]. The deliberately permissive primer combination used here allowed a wide range of mammalian DNA to be amplified within a single reaction, although this meant that some identification did not go beyond the level of genus. For example, no. 25029 was identified as *Canis* but did not distinguish between *Canis lupus* (wolf), *Canis latrans* (coyote) and *Canis domesticus* (domestic dog).

Sequences derived from hair sample nos. 25025 and 25191 had a 100% match with DNA recovered from a Pleistocene fossil more than 40 000 BP of *U. maritimus* (polar bear) [12] but not to modern examples of the species. Hair sample no. 25025 came from an animal shot by an experienced hunter in Ladakh, India *ca* 40 years ago who reported that its behaviour was very different from a brown bear *Ursus arctos* with which he was very familiar. Hair sample no. 25191 was recovered from a high altitude (*ca* 3500 m) bamboo forest in Bhutan and was identified as a nest of a migyhur, the Bhutanese equivalent of the yeti. The Ladakh hairs (no. 25025) were golden-brown, whereas the hair from Bhutan (no. 25191) was reddish-brown in appearance. As the match is to a segment only 104 bp long, albeit in the very conserved 12S RNA gene, this result should be regarded as preliminary. Other than these data, nothing is currently known about the genetic affinity of Himalayan bears and although there are anecdotal reports of white bears in Central Asia and the Himalayas [13,14], it seems more likely that the two hairs reported here are from either a previously unrecognized bear species, colour variants of *U. maritimus*, or *U. arctos*/*U. maritimus* hybrids. Viable *U. arctos*/*U. maritimus* hybrids are known from the Admiralty, Barayanov and Chicagov (ABC) islands off the coast of Alaska though in the ABC hybrids the mitochondrial sequence homology is with modern rather than ancient polar bears [15]. If they are hybrids, the Ladakh and Bhutan specimens are probably descended from a different hybridization event during the early stages of species divergence between *U. arctos* and *U. maritimus.* Genomic sequence data are needed to decide between these alternatives. If these bears are widely distributed in the Himalayas, they may well contribute to the biological foundation of the yeti legend, especially if, as reported by the hunter who shot the Ladakh specimen, they behave more aggressively towards humans than known indigenous bear species.

With the exception of these two samples, none of the submitted and analysed hairs samples returned a sequence that could not be matched with an extant mammalian species, often a domesticate. While it is important to bear in mind that absence of evidence is not evidence of absence and this survey cannot refute the existence of anomalous primates, neither has it found any evidence in support. Rather than persisting in the view that they have been ‘rejected by science’, advocates in the cryptozoology community have more work to do in order to produce convincing evidence for anomalous primates and now have the means to do so. The techniques described here put an end to decades of ambiguity about species identification of anomalous primate samples and set a rigorous standard against which to judge any future claims.

## Data accessibility

DNA sequences: GenBank accession nos. KJ155696–KJ155724 and KJ607607. Voucher samples of the research materials have been deposited in the Heuvelmans Archive at the Museum of Zoology, Lausanne, Switzerland.
